# Research on Zheng Classification Fusing Pulse Parameters in Coronary Heart Disease

**DOI:** 10.1155/2013/602672

**Published:** 2013-04-30

**Authors:** Rui Guo, Yi-Qin Wang, Jin Xu, Hai-Xia Yan, Jian-Jun Yan, Fu-Feng Li, Zhao-Xia Xu, Wen-Jie Xu

**Affiliations:** ^1^Laboratory of Information Access and Synthesis of TCM Four Diagnostic, Center for TCM Information Science and Technology, Shanghai University of Traditional Chinese Medicine, Shanghai 201203, China; ^2^Center for Mechatronics Engineering, East China University of Science and Technology, Shanghai 200237, China

## Abstract

This study was conducted to illustrate that nonlinear dynamic variables of Traditional Chinese Medicine (TCM) pulse can improve the performances of TCM Zheng classification models. Pulse recordings of 334 coronary heart disease (CHD) patients and 117 normal subjects were collected in this study. Recurrence quantification analysis (RQA) was employed to acquire nonlinear dynamic variables of pulse. TCM Zheng models in CHD were constructed, and predictions using a novel multilabel learning algorithm based on different datasets were carried out. Datasets were designed as follows: *dataset1*, TCM inquiry information including inspection information; *dataset2*, time-domain variables of pulse and *dataset1*; *dataset3*, RQA variables of pulse and *dataset1*; and *dataset4*, major principal components of RQA variables and *dataset1*. The performances of the different models for Zheng differentiation were compared. The model for Zheng differentiation based on RQA variables integrated with inquiry information had the best performance, whereas that based only on inquiry had the worst performance. Meanwhile, the model based on time-domain variables of pulse integrated with inquiry fell between the above two. This result showed that RQA variables of pulse can be used to construct models of TCM Zheng and improve the performance of Zheng differentiation models.

## 1. Introduction

Traditional Chinese Medicine (TCM) has made great contribution worldwide. In long term medical practice, Chinese physicians have realized the close relationship between the external expressive form of the human body and the essence of diseases; they have diagnosed diseases through “Zheng differentiation.” The Zheng differentiation of TCM considers the etiology, location, nature, and condition of a disease, as well as the struggle between body resistance and pathogenic factors, during a specific stage of the disease process. Zheng is a unique TCM concept. It is an abstractive collection of various signs and symptoms and is a pathological summary of a specific stage during the course of a disease. The signs and symptoms can be captured by doctors through four diagnostic methods, namely, inspection, auscultation and olfaction, inquisition, and pulse taking. TCM pulse taking has been proven to be clinically valid for over 2000 years. During pulse taking, TCM doctors place their fingers on the radial artery, from which various physiological and pathological conditions can be detected. Traditional pulse taking has important clinical value on the diagnosis and prognosis of diseases, especially angiocardiopathy. Accurate pulse taking can only be done by TCM doctors after years of experience. Therefore, objective and digital pulse diagnosis is highly desirable.

For many years, the quantification problem of TCM pulse taking has been a hot subject in multidisciplinary research. Currently, numerous researchers in China and abroad have put forward different methods for pulse analysis, such as time-domain, frequency-domain, and time-frequency joint analyses [1–4]. However, these traditional methods based on linear concepts are restricted to system stability. They are not particularly sensitive to changes in pathological state and are insufficient in characterizing the complex dynamics of a nonlinear system. Nonlinear dynamics methods for time series prediction can obtain nonlinear information on pulse, which cannot be achieved by traditional analytical methods. These nonlinear dynamics methods have wide applications in physiological signals, such as heart rate variability, electroencephalogram, electrocardiogram, and electromyography [5–7].

Numerous methods (e.g., K-S entropy, correlation dimension, the largest Lyapunov exponent, etc.) that can disclose nonlinear characteristics of physiological signals have been established. However, these aforementioned methods require large data and are susceptible to noise. Actual measured physiological signals often fail to meet these requirements. To overcome these problems, Eckmann et al. [8] introduced a tool that enables us to investigate the *m*-dimensional phase space trajectory through a two-dimensional representation of its recurrences. This representation is called a recurrence plot (RP). RPs can also be applied to rather short and even nonstationary data. Webber and Zbilut [9, 10] developed recurrence quantification analysis (RQA), a tool that quantifies structures in RPs. Marwan et al. [11, 12] supplemented RQA variables to quantify RPs. In the present study, RQA was employed to acquire the nonlinear dynamic features of the wrist pulse of coronary heart disease (CHD) patients and normal subjects. For Zheng pattern classification, RQA variables of pulse in CHD patients were analyzed.

Zheng is the pathological summary of a disease during a certain stage. The symptoms constituting both Zheng and disease are the most essential elements for disease identification and Zheng differentiation. The different stages of a disease may have various Zheng, and a similar Zheng may occur in other diseases. Deficiency and excess are the two main principles in differentiating the preponderance or decline of pathogenic factors and healthy-qi. Deficiency Zheng and excess Zheng can reflect the two aspects of struggle between body resistance and pathogenic factors during a disease process. The former refers to deficiency of body resistance, whereas the latter refers to excessive and hyperactivity of pathogenic factors. In clinical practice, the Zheng of mixed deficiency and excess, which refers to a complex condition of concurrent deficiency and excess, is common. For example, the pattern of heart-qi deficiency (deficiency Zheng) in CHD patients is accompanied by the obstruction pattern of heart vessels by phlegm (excessive Zheng). Thus, one case is often associated with multiple Zheng patterns. Therefore, TCM Zheng patterns can be classified as a multi-label problem that traditional single-label learning algorithms cannot solve. The multi-label learning (MLL) algorithm REAL is suitable for solving multi-label recognition of TCM Zheng patterns [13]. This algorithm was applied to construct Zheng classification models based on different datasets. The performances of models were compared.

The remainder of the paper was organized as follows. In Section 2, we described the collection of clinical data, including inquiry, inspection, and pulse. In Section 3, we introduced the pulse data processing method through RQA, the recognition model of Zheng pattern-based REAL, and the evaluation measures for the recognition performance of the Zheng model. In Section 4, we summarized the results. A statistical analysis of RQA variables was made between CHD patients and normal subjects to establish the physiological and pathological significance of RQA variables. Zheng classification models of CHD were constructed based on REAL using different datasets. The datasets were designed as follows: *dataset1*, inquiry and inspection information; *dataset2*, time-domain variables integrated with *dataset1*; *dataset3*, RQA variables of pulse integrated with *dataset1*; and *dataset4*, major principal components of RQA variables and *dataset1.* Moreover, the recognition performances of different Zheng models were compared according to evaluation measures, such as *average precision*, *overage*, *hamming loss*, and *one error,* and *rank loss*. In Section 5, we discussed the effect of the RQA variables of pulse on the recognition performance of TCM Zheng in CHD patients. 

## 2. Material

### 2.1. Collected Material

Patients who met the diagnostic criteria of CHD and those who provided informed consent were included in the present study. The diagnostic criteria of patients were based on western medicine and TCM. The diagnostic criteria based on western medicine was adopted from “naming and diagnosis criteria of ischemic heart disease” issued by the International Society of Cardiology and the Joint Subject Team on standardization of clinical naming in the World Health Organization [14]. The diagnostic criteria based on TCM were according to the “differentiation standards for symptoms and signs of coronary heart disease and angina pectoris in Traditional Chinese Medicine” in the “standards for differentiation of chest pain, chest distress, palpitation, short breath, or debilitation for coronary heart disease in Traditional Chinese Medicine” modified by the China Society of Integrated Traditional Chinese and Western Medicine in 1990, the “Guideline for clinical study of new drugs in Chinese herbs,” and the standards in textbooks [15]. Patients with mental diseases or other severe diseases, as well as those who cannot express their feelings clearly and did not provide informed consent, were excluded in the present study.

456 CHD cases including pulse recordings were collected. According to the inclusion and exclusion criteria of the patients, 334 CHD cases were included, and 121 CHD cases were excluded in this study. Pulse recordings of 117 normal subjects (control group) were also collected. The CHD patients (age, 63.00 ± 10.74) were those admitted to Longhua Hospital and Shuguang Hospital, which are affiliated to Shanghai University of Traditional Chinese Medicine. The healthy subjects (age, 52.17 ± 11.00) were players of the “2010 Zhangjiang ball game competition for the elderly” and the faculty of the Shanghai University of Traditional Chinese Medicine. The CHD patients were clinically differentiated using Zheng patterns by integrating inspection, pulse feeling, and inquiry information. Each of the 334 patients has more than one Zheng pattern and occurred mostly in the pattern of heart-qi deficiency (172 cases), heart-yang deficiency (72 cases), heart-yin deficiency (219 cases), and heart-vessel obstruction by phlegm (170 cases). Therefore, these four Zheng patterns were selected in this study. The pattern of heart -qi deficiency refers to the weakness of heart-qi to pump blood, that is, blood circulation. Its main symptoms include palpitations, mental fatigue, and general symptoms of qi deficiency Zheng. The pattern of heart-yang deficiency refers to the failure of heart-yang to warm and circulate blood and to the internal production of deficient cold. Its main symptoms include palpitations, chest oppression, and general symptoms of deficient cold Zheng. The pattern of heart-yin deficiency refers to the failure of yin-fluid consumption to nourish the heart and heart-mind and to the internal disturbance of deficient-heat. Its main symptoms include palpitations, restlessness, insomnia, and general symptoms of deficient-heat Zheng. The pattern of heart-vessel obstruction by turbid phlegm refers to the condition of turbid-phlegm obstructing the heart vessel. It is mainly include mild or severe palpitation, chest oppression, and pain.

### 2.2. Acquisition of Inquiry and Inspection Information

In this study, inquiry symptoms were quantified according to the scale of inquiry diagnosis for the heart system [16]. The symptoms collected for inquiry diagnosis involve temperature, sweating, head, body, chest and abdomen, urine and stool, appetite, sleeping, mood, gynecology, tongue color, and face color, a total of 125 symptoms. The symptom was assigned with either “1” or “0,” referring to “existence” or “nonexistence,” respectively. The symptoms were removed when their frequencies of existence were not more than 10 to avoid interference of data redundancy. Thus, a total of 79 symptoms were selected for Zheng modeling in CHD.

### 2.3. Acquisition of Pulse Recordings

Pulse recordings were acquired using a Z-BOX type pulse measurement device and classical time-domain variables *h*_1_, *h*_3_, *h*_4_, *h*_5_, *t*_1_, *t*_4_, *t*_5_, *t*, *w*, *h*_3_/*h*_1_, *h*_4_/*h*_1_, *h*_5_/*h*_1_, *w*/*t*, *A*_*s*_, and *A*_*d*_ were calculated using the software included in the Z-BOX type pulse measurement device. Each subject was asked to relax for more than 3 min before pulse acquisition. The pulse waveforms of all subjects were captured for 60 s at a sampling rate of 720 Hz. 

**Figure 1:**
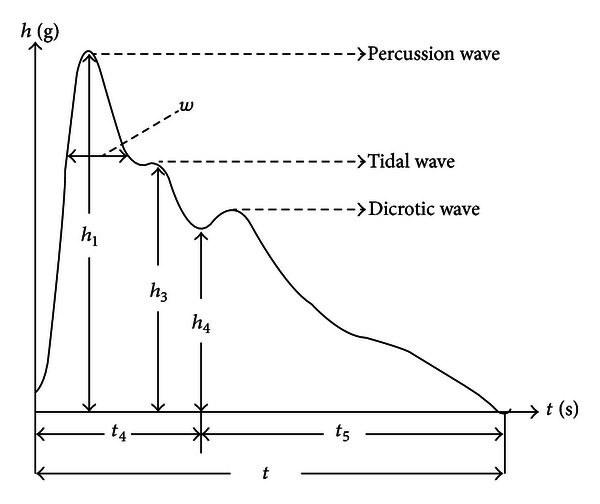
Height and time variables of a typical pulse cycle. *h*_1_: height of percussion wave, *h*_3_: height of tidal wave, *h*_4_: height of dicrotic notch, *t*_4_: time distance between the starting point of pulse chart and dicrotic notch, *t*_5_: time distance between dicrotic notch and the ending point of pulse chart, *t*: time distance between the starting point and the ending point, *w*: width of percussion wave in its 1/3 height position.

**Figure 2:**
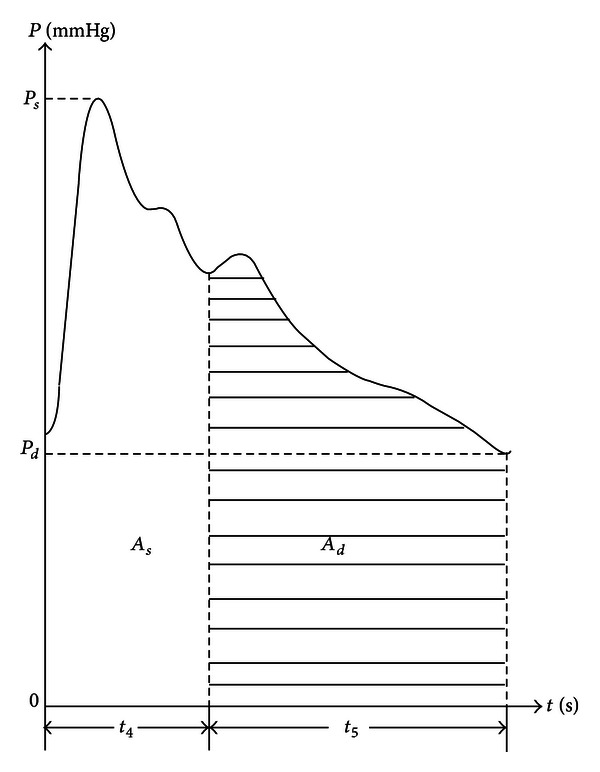
Area variables of a typical pulse cycle. *P*_*s*_: systolic pressure, *P*_*d*_: diastolic pressure, *A*_*s*_: area of contraction phase, and *A*_*d*_: area of diastolic phase.

 The time-domain method is most commonly used in pulse waveform analysis in TCM. This method has many applications in clinical practices. The classical time-domain variables are described through some characteristic points on the pulse wave (Figures 1 and 2) [17]. 

## 3. Methods

### 3.1. Extraction of RQA Variables of Pulse

**Figure 3:**
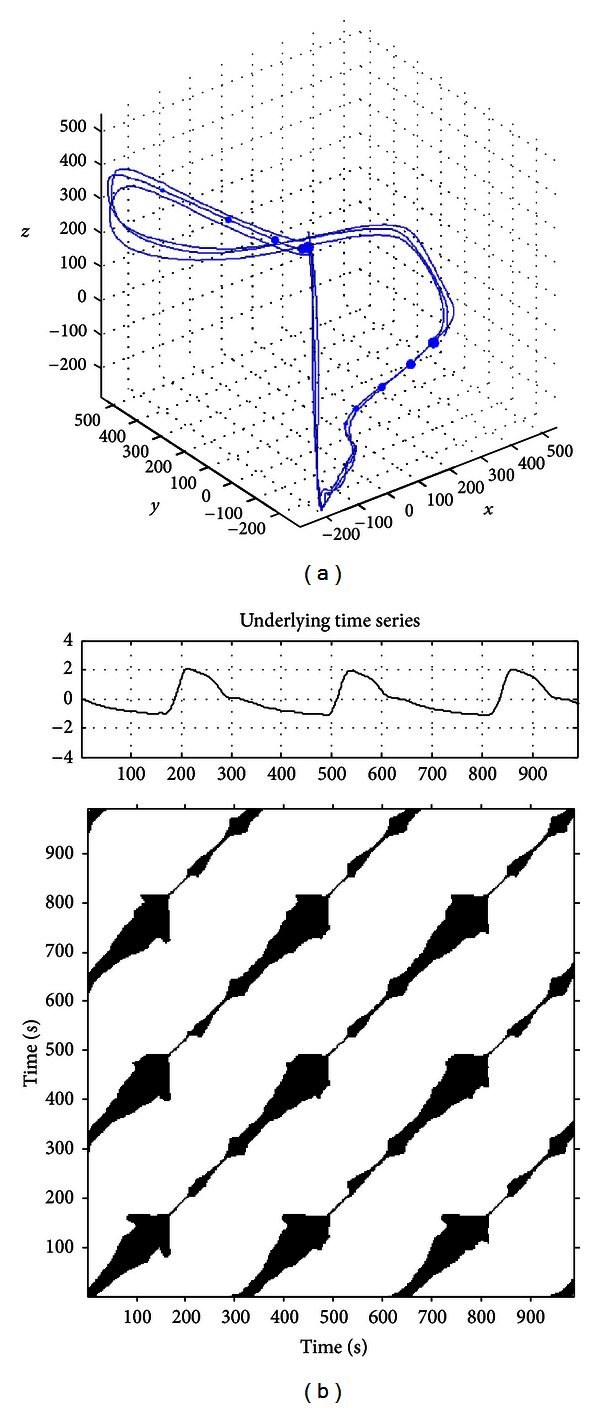
Reconstructed phase spaces and corresponding RP of the pulse recording of a CHD patient. (a) A segment from phase space trajectory of the pulse recording of a 60-year-old patient (*m* = 3,  *τ* = 5,  *ε* = 0.2*σ*, *σ* represents the variance of time series, *y* = *x* + *τ*,  and  *z* = *x* + 2∗*τ*). (b) Corresponding RP of (a).

Phase space reconstruction is the basis for the nonlinear time series analysis. It can be used to estimate the characteristic of the dynamic system. Usually, the phase space has to be reconstructed from the original one-dimensional time series [18, 19]. The time delay method is frequently used for reconstruction. For one-dimensional time series of length *M*, a trajectory *X*_*i*_ was reconstructed as *X*_*i*_ = (*x*(*i*),*x*(*i*+*τ*),…,*x*(*i*+(*m*−1)*τ*))^*T*^. The length of *X*_*i*_ was *N* = *M* − (*m* − 1) × *τ*, where the embedding dimension *m* can be estimated with the method of false nearest neighbors [20, 21], and the time delay *τ* can be estimated with the method of mutual information [22]. All these marked time trajectories {*X*_*i*_, *i* = 1,2,…, *N*} made up the *m*-dimension phase space orbits of the system.  Figure 3(a) showed an example of phase space reconstruction of one-dimensional pulse data. 

 RPs are a two-dimensional squared matrix with black and white dots, where black dots mark a recurrence and both axes were time axes Figure 3(b) [12]. RPs visualize the recurrent behavior of dynamical systems and can be mathematically expressed as [11, 12]

(1) $$\begin{array}{c} R_{i,j}=\Theta \left(\varepsilon_{i}-||{\overset{⃑}{x}}_{i}-{\vec{x}}_{j}||\right),\;i,j=1,2,\ldots ,N, \end{array}$$

where *N* is the number of considered states *x*_*i*_, *ε*_*i*_ is a threshold distance, ||·|| is a Euclidean norm, and Θ(·) is the Heaviside function.

 As shown in Figure 3, RP was composed of dots and lines in the diagonal and vertical structures. The diagonal structures mean that the evolution of states is similar at different times and that the process can be deterministic. The vertical structures describe the stability of the system. The visual interpretation of RPs only makes a qualitative analysis on the dynamic characteristics of the system. Thus, quantitative analysis of RPs was developed. Webber and Zbilut [9, 10] defined the measures of complexity using the recurrence point density and diagonal structures in the RPs. Gao [23] defined the measures of vertical structures. In the present study, we calculated the RQA measures of pulse using the following variables: recurrence rate (RR), determinism (DET), average diagonal line length (*L*), maximum diagonal structure length (*L*_max⁡_), Shannon entropy of the frequency distribution of diagonal line length (ENTR), laminarity (LAM), the average length of vertical structures (TT), and the maximal length of vertical structures (*V*_max⁡_).

Pulse morphology refers to blood pressure, vascular resistance, artery compliance, and so forth, which are important variables in assessing the cardiovascular system. Pulse morphological variation refers to the alterations and variations in the morphology of pulse waveform [24]. In this study, RQA was used to analyze pulse morphological variation. In this case, embedding dimension *m* = 3, time delay *τ* = 5, and distance cutoff *ε* = 0.3. Each pulse recording was segmented using a moving window with a size of 1000 sampling points. The window moves forward by 300 sampling points each time. Here, we applied eight RQA measures: {RR, DET, *L*, *L*_max⁡_, ENTR, LAM, TT, and *V*_max⁡_}. For each RQA measure during window movement, each pulse recording should produce a series of window RQA variables. The mean of the window RQA variables (MWRQA) and standard deviation of the window RQA variables (SWRQA) for each RAQ measure were calculated based on the following mathematical expressions:

(2) $$\begin{array}{c} \text{MWRQ}{\text{A}}_{j}=\frac{1}{\text{NW}}\sum_{i=1}^{\text{NW}}\text{RQ}{\text{A}}_{i}^{j},\\ \text{SWRQ}{\text{A}}_{j}=\sqrt{\frac{1}{\text{NW}}\sum_{i=1}^{\text{NW}}{\left(\text{RQ}{\text{A}}_{i}^{j}-\text{MWRQA}\right)}^{2}}, \end{array}$$

where NW is the number of windows, RQA_*i*_^*j*^ is the *j*th RQA variables of the *i*th windows of one pulse recording, MWRQA_*j*_ is the mean of the *j*th RQA variables of all windows of one pulse recording, and SWRQA_*j*_ is the standard deviation of *j*th RQA variables of all windows of one pulse recording. 

In this study, we selected eight RQA measures and then applied eight MWRQAs and eight SWRQAs to describe the morphological variation of a pulse recording. 

### 3.2. Construction of a Zheng Classification Model Using REAL

Multi-label learning deals with objects having multiple labels simultaneously. The TCM Zheng pattern belongs to such a problem. Formally, let *χ* = *ℜ* ^*d*^ be the *d*-dimensional input space, and let *y* = {*l*_1_, *l*_2_,…, *l*_*q*_} be the finite set of *q*  possible labels. The task of multi-label learning (or multi-label classification) is to learn a function *h* : *χ* → 2^*y*^ that maps each instance *x* ∈ *χ* into a set of proper labels *h*(*x*)⊆*y* [25]. REAL, a new multi-label leaning algorithm with features selected through the maximization of mutual information, was proposed by our research team and was confirmed suitable for TCM Zheng differentiation [13, 26]. For this method, feature variables associated mostly with Zheng were selected according to maximization of mutual information. This method fully paid attention to the relationship between features and Zheng; thus, it is more suitable for TCM Zheng classification. In this paper, REAL was applied to construct a model of TCM Zheng based on different datasets.

To evaluate the performance of Zheng models based on different datasets, five evaluation measures [27, 28] especially designed for multi-label learning were used.

*Average precision* evaluates the average fraction of proper labels ranked above a particular label set. For this evaluation measure, higher values mean better classifier performance. 

*Coverage* evaluates how far we need, on the average, to go down the list of labels to cover all the proper labels of the multi-label example. For this evaluation measure, smaller values mean better performance.

*Hamming loss* evaluates how many times an example label pair was misclassified. For this evaluation measure, smaller values of hamming loss mean better performance.

*One error* evaluates how many times the top-ranked label was not in the set of proper labels of the multi-label example. For this evaluation measure, smaller values mean better performance.

*Ranking loss* evaluates the average fraction of Zheng label pairs that were disordered for the multi-label example. For this evaluation measure, smaller values of ranking loss mean better performance.

## 4. Results

### 4.1. Comparison of RQA Variables of Pulse between the CHD Patients and Normal Subjects

The physiological and pathological significances of RQA variables were discussed in Section 5. In this section, a statistical analysis of pulse RQA variables using independent sample *t*-test and rank-based ANOVA was made between the CHD patients and healthy subjects. Independent sample *t*-test was applied to analyze the RQA variables with normal distribution and homogeneous variance. Rank-based ANOVA was applied for analysis of RQA variables without these aforementioned requirements. Age variable as covariant was included in the statistical model to correct the effect of age. In Table 1, *P* values of group effects were calculated after age effects were corrected. As shown in Table 1, the RQA variables of two groups, except for the MWRQAs of *L*, SWRQAs of LAM, and SWRQAs of DET, had not significantly difference. The MWRQAs of {RR, DET, *L*_max⁡_, ENIR, LAM, TT, and *V*_max⁡_} and SWRQAs of {RR, *L*, *L*_max⁡_, ENIR, TT, and *V*_max⁡_} of the CHD patients were significantly higher than those of the healthy subjects. 

### 4.2. Principal Components Analysis of RQA Variable

Traditionally, principal component analysis (PCA) has been the standard approach to reduce the high-dimensional original pattern vector space into low-dimensional feature vector space. It has wide applications in feature identification. RQA variables contained much redundancy because some variables may be highly dependent on each other. In the present study, PCA extracted four major principal components of RQA variables that represent 83.24% of those that RQA variables can represent, which may be very useful for purpose of classification. 

### 4.3. Comparison of Model Performance for Zheng Differentiation with/without RQA Variables

In this study, each CHD case was simultaneously associated with multiple Zheng, namely, Heart-qi deficiency, heart-yang deficiency, heart-ying deficiency, and heart-vessel obstruction by turbid phlegm. Thus, Zheng classification models were constructed, and predictions were made using the MLL algorithm REAL. For comparison, datasets for the construction of the models were designed as follows: (1)  *dataset1*, only inquiry and inspection information; (2)  *dataset2*, time-domain variables of pulse and *dataset1*; (3) *dataset3*, RQA variables of pulse and *dataset1*; and (4) *dataset4,* the four major principal components of RQA variables and *dataset1*. A tenfold cross-validation was performed on each experimental dataset. Up to 90% of the samples were randomized as the training set; the other 10% were randomized as the test set. Prediction analysis for TCM Zheng was performed after re-testing the models 10 times and taking the mean value. 

The performances of the different models based on different datasets were compared in terms of different evaluation measures (Table 2). For *average precision*, higher values meant better performance. For the other four evaluation measures (*coverage*, *hamming loss*, *one error*, and *rank loss*), smaller values meant better performance. As shown in Table 1, for *dataset1*, the value of *average precision* was the lowest. The values of the other four evaluation measures were the highest. These results showed that the performance of the model based on *dataset1* without pulse information was the worst. For *datset2* with time-domain variables of pulse, the performance of the model was superior to that in *dataset1* but was inferior to those in *dataset3 and dataset4* including information that RQA variables of pulse can represent. The model based on *dataset4* had the best performance owing to major principal component of RQA variables that maintain the correlated part of information discarding the minor noise-dominated part of information, which can improve the recognition ability of the model.

## 5. Discussion

Pulse diagnosis is one of the most important diagnostic methods in TCM. The pulse driven by the heart propagates through the arteries. Hence, the pulse wave should contain most of the information about the cardiovascular system. Many clinical experiments have confirmed that pulse condition is closely related to the cardiovascular system. The pulse wave form (shape), velocity (fast or slow), period (rhythm), and swing (intensity) are affected by the physiological and pathological features of the cardiovascular system [29]. The traditional pulse analytical methods include time-domain, frequency-domain, and time-frequency joint analyses. However, these methods based on linear concepts cannot be used to extract nonlinear features of the pulse. Therefore, in this study, the nonlinear dynamics method RQA was applied to extract nonlinear features of the pulse. RQA represents recurrence phenomenon, a fundamental property of deterministic dynamic systems. Different RQA variables represent different nonlinear dynamic characteristics. RR is a measure of recurrence density. High RR values indicate the presence of a strong cyclical process. *L* and DET are measures of system determinism. High *L* and DET values suggest stronger system determinism. ENTR is a measure of the complexity of the deterministic structure in the system. LAM, TT, and *V*_max⁡_ mark a time interval, wherein a state does not change or changes very slowly. Therefore, higher LAM, TT, and *V*_max⁡_ values indicate higher system stability. As shown in Table 1, the MWRQAs of {RR, DET, *L*, *L*_max⁡_, ENIR, LAM, TT, and *V*_max⁡_} of the CHD patients were significantly higher than those of the healthy subjects, and the mean *L* of the healthy subjects was slightly higher than that of the CHD patients. This finding indicates that the cardiovascular system of the CHD patients, closely connected with pulse, had greater regularity and stability than that of the normal subjects, which is consistent with the discovery by many researches that system in pathological state is usually more stable. Moreover, the SWRQAs of {RR, *L*, *L*_max⁡_, ENTR, LAM, TT, and *V*_max⁡_} of the CHD patients were significantly higher than those of the healthy subjects. This finding suggests that the pulse morphology of the CHD patients has larger variability than that of the normal subjects. Hence, we speculate that the CHD patients show weaker ability in regulating the cardiovascular system than the healthy subjects.

CHD is a complex process expressed as different Zheng patterns in its different stages. CHD can disrupt the pumping function of the heart, causing changes in both the structure and function of blood vessels and inevitably giving rise to changes in pulse. These changes vary with different Zheng patterns. Therefore, in this study, the RQA variables of pulse were used to characterize the changes in Zheng classification. In clinical practice, one patient is often associated with several Zheng patterns. Zheng classification belongs to a multi-label problem. Therefore, Zheng models were constructed, and predictions were carried out using MLL algorithm REAL. The performances of models based on different datasets were compared (Table 2). According to the five evaluation measures, the model for Zheng differentiation based on RQA variables integrated with inquiry had the best performance, whereas that based only on inquiry information had the worst performance. Meanwhile, the model based on time-domain variables of pulse integrated with inquiry fell between the above two. Therefore, the RQA variables can improve the ability of a model for Zheng classification. Moreover, the model based on *dataset4* had the best performance owing to major principal component of RQA variables that maintain the correlated part of information discarding the minor noise-dominated part of information, which can improve the recognition ability of the model.

The linear method represented by the classical time-domain method is widely used to analyze pulse. However, the nonlinear dynamics method looks bright and promising in revealing the inside information and dynamic properties of systems. The RQA variables of pulse can be used to construct models of TCM Zheng and improve the performance of Zheng differentiation models. 

## Figures and Tables

**Table 1:** RQA variables show significant differences, as determined by independent sample *t*-test and rank-based ANOVA.

Measures	Groups		*P*
	Patient with CHD	Normal subjects	
MWRQAs* of *RR	0.07 ± 0.0141^∗□^	0.0624 ± 0.008^□^	*P* = 0.015
MWRQAs* of *DEI	0.998 ± 0.0018^∗□^	0.993 ± 0.0102^□^	*P* < 0.001
MWRQAs* of L *	28.714 ± 8.582^□^	25.201 ± 7.148^□^	*P* = 0.198
MWRQAs* of L*_max⁡_	557.941 ± 257.692^∗□^	400 ± 197.999^□^	*P* < 0.001
MWRQAs* of *ENTR	4.036 ± 0.275^∗□^	3.893 ± 0.285^□^	*P* = 0.044
MWRQAs* of *LAM	0.996 ± 0.0021^∗□^	0.995 ± 0.0034^□^	*P* = 0.001
MWRQAs* of *TT	16.606 ± 3.980^∗□^	13.947 ± 2.587^□^	*P* = 0.001
MWRQAs* of V*_max⁡_	70.616 ± 18.727^∗□^	55.198 ± 9.759^□^	*P* = 0.001
SWRQAs* of *RR	0.011 ± 0.005^∗□^	0.007 ± 0.003^□^	*P* < 0.001
SWRQAs* of *DET	230 (102.5–336.5)^△^	210 (131–358)^△^	*P* = 0.102
SWRQAs* of L *	4.574 ± 1.936^∗□^	3.316 ± 1.443^□^	*P* < 0.001
SWRQAs* of L*_max⁡_	138.739 ± 89.413^∗□^	94.483 ± 90.881^□^	*P* < 0.001
SWRQAs* of *ENTT	0.165 ± 0.087^∗□^	0.133 ± 0.002^□^	*P* < 0.001
SWRQAs* of *LAM	236 (106.5–347)^△^	219 (113.75–331)^△^	*P* = 0.362
SWRQAs* of *TT	2.208 ± 1.165^∗□^	1.386 ± 0.602^□^	*P* < 0.001
SWRQAs* of V*_max⁡_	15.383 ± 9.035^∗□^	7.788 ± 4.126^□^	*P* < 0.001

Compared with normal group, *difference was significant. ^□^Analyzed by independent sample *t*-test (mean ± standard deviation); ^△^analyzed by rank-based ANOVA (M (QL–QU)).

**Table 2:** Performance comparison of Zheng classification models based on different datasets.

Group	Dataset1	Dataset2	Dataset3	Dataset4 (PCA)
*Average precision *	0.8423	0.8534	0.8626	0.8735
*Coverage *	0.3932	0.3925	0.3786	0.3604
*Hamming loss *	0.2621	0.2684	0.2428	0.2228
*One error *	0.2454	0.2089	0.1996	0.1982
*Rank loss *	0.1944	0.1875	0.1768	0.1543

*Note: dataset1*, inquiry information; *dataset2*, time-domain variables of pulse and dataset1; *dataset3*, RQA variables of pulse and dataset1; *dataset4*, four major principal components of RQA variables and *dataset1*.
